# Switchable Metal Sites in Metal–Organic Framework MFM‐300(Sc): Lewis Acid Catalysis Driven by Metal–Hemilabile Linker Bond Dynamics

**DOI:** 10.1002/anie.202210857

**Published:** 2022-10-25

**Authors:** Ricardo A. Peralta, Pengbo Lyu, Alfredo López‐Olvera, Juan L. Obeso, Carolina Leyva, Nak Cheon Jeong, Ilich A. Ibarra, Guillaume Maurin

**Affiliations:** ^1^ Department of Physics & Chemistry Center for Basic Science, DGIST Daegu 42988 Korea; ^2^ Departamento de Química, División de Ciencias Básicas e Ingeniería, UAM-I 09340 México Mexico; ^3^ ICGM Univ. Montpellier, CNRS ENSCM Montpellier 34095 France; ^4^ Hunan Provincial Key Laboratory of Thin Film Materials and Devices School of Material Sciences and Engineering Xiangtan University Xiangtan 411105 China; ^5^ Laboratorio de Fisicoquímica y Reactividad de Superficies (LaFReS). Instituto de Investigaciones en Materiales Universidad Nacional Autónoma de México Circuito Exterior s/n, CU, Coyoacán 04510 Ciudad de México Mexico; ^6^ Instituto Politécnico Nacional CICATA U. Legaria 694 Irrigación 11500 Miguel Hidalgo, CDMX México Mexico

**Keywords:** Catalysis, Density Functional Theory, MOFs, Metal-Hemilabile Linker Bond Dynamics, Reaction Mechanism

## Abstract

Uncommon reversible guest‐induced metal‐hemilabile linker bond dynamics in MOF MFM‐300(Sc) was unraveled to switch on/switch off catalytic open metal sites. The catalytic activity of this MOF with non‐permanent open metal sites was demonstrated using a model Strecker hydrocyanation reaction as a proof‐of‐concept. Conclusively, the catalytic activity was evidenced to be fully reversible, preserving the conversion performance and structure integrity of MFM‐300(Sc) over multiple cycles. These experimental findings were corroborated by quantum‐calculations that revealed a reaction mechanism driven by the Sc‐open metal sites. This discovery paves the way towards the design of new effective and easily regenerable heterogeneous MOF catalysts integrating switchable metal sites.

## Graphical abstract

An unconventional catalytic activity was demonstrated for the Strecker reaction using a saturated Sc^III^‐based MOF known as MFM‐300(Sc). This activity was possible due to metal‐hemilabile linker bond to create an intermittent free Sc^III^ site.

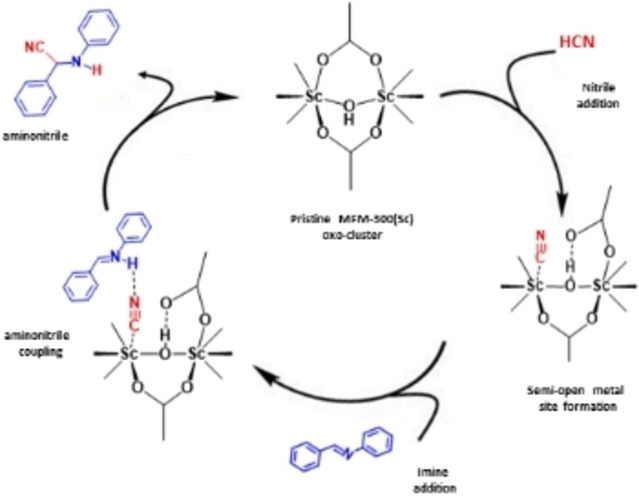

## Introduction

Metal–Organic Frameworks (MOFs) combine stability, confinement and matrix‐isolation of heterogeneous catalysts with molecular tunability of homogeneous catalysts.^[1]^ This family of hybrid porous materials have demonstrated to successfully achieve a wide variety of catalytic transformations.[^2^, ^3^, ^4^, ^5^, ^6^, ^7^, ^8^, ^9^] The catalytic sites in MOFs are (*i*) “metallolinkers”, i.e., multi‐variant organic molecules that bear catalytically active metal sites while also serving as building units of the framework,^[10]^ (*ii*) cluster‐based metal ions that are also connected to the organic linkers to form the architecture of MOFs,^[11]^ or (*iii*) metal ions/clusters appended to the inorganic and/or organic nodes.^[12]^ These metal sites, commonly named open metal sites (OMS) are prone to form a strong metal‐reactant bond for initiating the catalytic reaction. Making OMS of MOFs accessible to adsorbed chemical species is mostly achieved by removing still‐coordinated solvent molecules (water, alcohol and DMF),^[13]^ creating defective linkers^[14]^ or using post‐synthetic metalations.^[15]^ The first approach calls for a well‐controlled activation procedure implying most often a thermal treatment that hardly leads to a full evacuation of OMS while it can alter the structure (at least locally) or even the reactivity of these sites (change on the oxidation state). Although many efforts have been approached over the last few years for a better control of linker defects in various MOFs,^[16]^ metal sites are still capped by terminal groups (−OH, −H_2_O, solvents, and modulators) and the identification of the real catalytic active site remains sometimes elusive. Finally, assembling MOFs with organometallic species in the linker often leads to metallo‐linker decomposition or ligand redistribution within the MOF.^[17]^ On the other hand, post‐synthetic metalation risks the metal ions appending to unintended sites of the MOF framework, such as bridging inorganic oxo units.^[12]^ Therefore this overall observation emphasizes the need to find alternative strategies to design MOF catalysts that do not necessarily contain permanent OMS.

**Figure 1:**
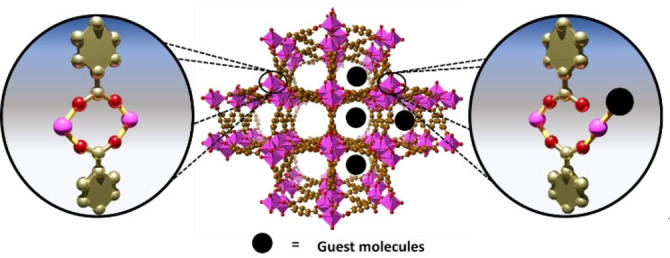
Illustration of the metal‐hemilabile ligand bond dynamics upon guest adsorption in MFM‐300(Sc). (C, gold; O, red; Sc, pink polyhedron; Guest molecule, black).

The concept of hemilability in MOFs and different consequences on the hydrolytic stability and many other properties of this family of porous materials, it has been intensively developed by Morris et al.[^18^, ^19^, ^20^] The associated reversible metal‐ligand bond dynamics can explain key phenomena in MOFs from their crystal growth to their phase transitions.^[21]^ For example, Bennett and Horike, elegantly proposed, the different melting mechanisms for ZIFs.^[22]^ This reversible metal‐ligand bond dynamics was shown to be triggered by temperature but also by guest‐adsorption.^[23]^ We recently^[24]^ demonstrated that NH_3_ adsorption induces a reversible metal‐linker bond rearrangement in MFM‐300(Sc)^[25]^ ([Sc_2_(biphenyl‐3,3′,5,5′‐tetracarboxylate)(μ‐OH)_2_) resulting from a labile scandium‐carboxylate bond (Figure 1),^[24]^ that enables a strong‐binding of NH_3_ towards the created Sc^III^ OMS.^[21]^ Previously, another study evoked the potential implication of this metal‐ligand dynamics in the field of catalysis with the cyclohexene oxidation by MIL‐47(V) containing chains of corner‐sharing fully coordinated V^4+^O_6_ octahedra interconnected by 1,4‐dicarboxylate linkers.^[26]^

Inspired by our previous findings on the Sc−O bond dynamics in MFM‐300(Sc), herein we deliberately explore the ability of this MOF to act as a catalyst for the so‐called Strecker hydrocyanation reaction.^[27]^ This reaction is usually carried out in homogeneous systems and proceeds via the nucleophilic addition of a cyanide ion to the imines using different Lewis acid catalysts. This model reaction has been thus selected as a show‐case since it was demonstrated that the Lewis acid Sc^III^ metal, among others such as Y^III^ ^[24]^ and Ln^III^ ^[28]^ and In^III^,^[29]^ plays an essential role in the synthesis of R‐aminonitriles via an effective C=N_imine_ bond activation throughout a Sc^III^‐substrate coordination. It was previously demonstrated that this kind of reaction can be carried out via heterogeneous catalysis with the aid of MOFs.^[30]^ The pioneer work reported by Monge and Gándara evidenced that the InPF‐110 MOF containing In^III^ OMS is catalytically active to produce 2‐Phenyl‐2‐(phenylamino)propanenitrile from acetophenone, aniline and trimethylsilyl cyanide (TMSCN) by a Strecker reaction.^[31]^

**Scheme 1:**
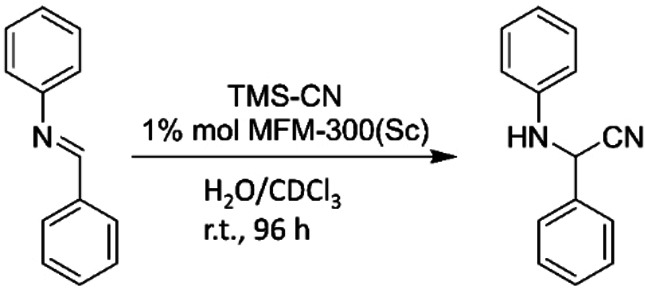
Strecker reaction catalyzed by MFM‐300(Sc).

Herein, our experimental exploration on MFM‐300(Sc) as a potential catalyst for the formation of 2‐phenyl‐2‐(phenylamino)acetonitrile from the reaction between N‐Benzylideneaniline and TMSCN (scheme 1) was complemented by Density Functional Theory (DFT) calculations. We aimed to gain mechanistic insight on the catalytic reaction with the ultimate goal to demonstrate that the use of hemilabile linkers offers an optimum avenue to reversibly switch on/switch off the active sites of a MOF catalyst. This MOF catalyst engineering strategy presents several advantages; (*i*) the organic linkers can spontaneously associate/dissociate from the inorganic nodes without affecting the structural integrity of the material, (*ii*) the intermittent existence of the OMS created only once the reactant is introduced makes unnecessary the fastidious optimization of the activation protocol usually applied to OMS‐containing MOFs and (*iii*) a reversible reactant‐triggered switch‐ability of the active sites from a full coordination towards OMS, is expected to enable selected examples of MOFs to retain their full catalytic activity over cycling and an easy regeneration. This final point was confirmed by performing several Strecker reaction cycles with MFM‐300(Sc). These overall findings can pave the way towards the design of heterogeneous MOF‐based catalysis easily regenerable and maintaining high‐level performance over cycling.

## Results and Discussion

MFM‐300(Sc) was synthesized according to the literature^[25]^ and powder X‐ray diffraction (PXRD) confirmed the phase purity of the resulting material (Figure S1). A MOF sample was acetone‐exchanged and then heated up at 180 °C and 1.7×10^−3^ torr for 6 h, leading to a BET area of 1385 m^2^ g^−1^ and a pore volume of 0.56 cm^3^ g^−1^ (Figure S2) in excellent agreement with the previously reported values (1390 m^2^ g^−1^ and 0.58 cm^3^ g^−1^, respectively).^[25]^ This material was further tested as catalyst for the Strecker reaction between N‐Benzylideneaniline and TMSCN with wet chloroform‐d at room temperature (r.t) leading to the formation of 2‐phenyl‐2‐(phenylamino)acetonitrile (Scheme 1).

The synthesized MFM‐300(Sc) crystals were first soaked in chloroform‐d and exposed to N‐Benzylideneaniline and TMSCN for 96 hours at room temperature. The material was demonstrated to be catalytically active for this Strecker reaction with a conversion of about 45 %, which was monitored by ^1^H NMR. The key role played by MFM‐300(Sc) was confirmed by a control experiment performed, were its absence did not reveal any conversion. In parallel, the same reaction carried out in a homogeneous Sc^III^ chloroform‐d solution led to a conversion of 91 % in 20 h in line with the previously reported data collected under the same conditions.^[32]^ The lower activity exhibited by MFM‐300(Sc) as compared to an homogeneous catalysis is due to a less‐straightforward access to the Sc^III^ metal sites. It is also worth mentioning that the reaction conditions were not optimized since our objective was to prove the catalytic activity of this MOF without permanent OMS and not its level of performance. Nevertheless, this fascinating dynamic metal‐ligand bonding presented in MFM‐300(Sc) offers an excellent opportunity to heterogenized the Strecker reaction in a highly stable material to address the standard limitations of the homogeneous catalysis i.e., poor cyclability, hard recovery, low selectivity, poor tolerance to water, among others.^[30]^

It is relevant to mention that the reaction with TMSCN was done in CDCl_3_ in the presence of water (0.07 mol % maximum solubility of water in chloroform‐d at r.t) that generates HCN and TMSOH^[33]^ as detected by ^1^H NMR. Interestingly, under strictly anhydrous conditions, no catalytic reaction was observed, corroborating that HCN is the key intermediate species to initiate the reaction and that water is most likely the source of H^+^. In addition, we demonstrated that the reaction conversion gradually increases, as the amount of water present in the reaction raised, until the quantity of HCN as a byproduct was abundant enough to not modify the product yield (Figure S8).

**Figure 2:**
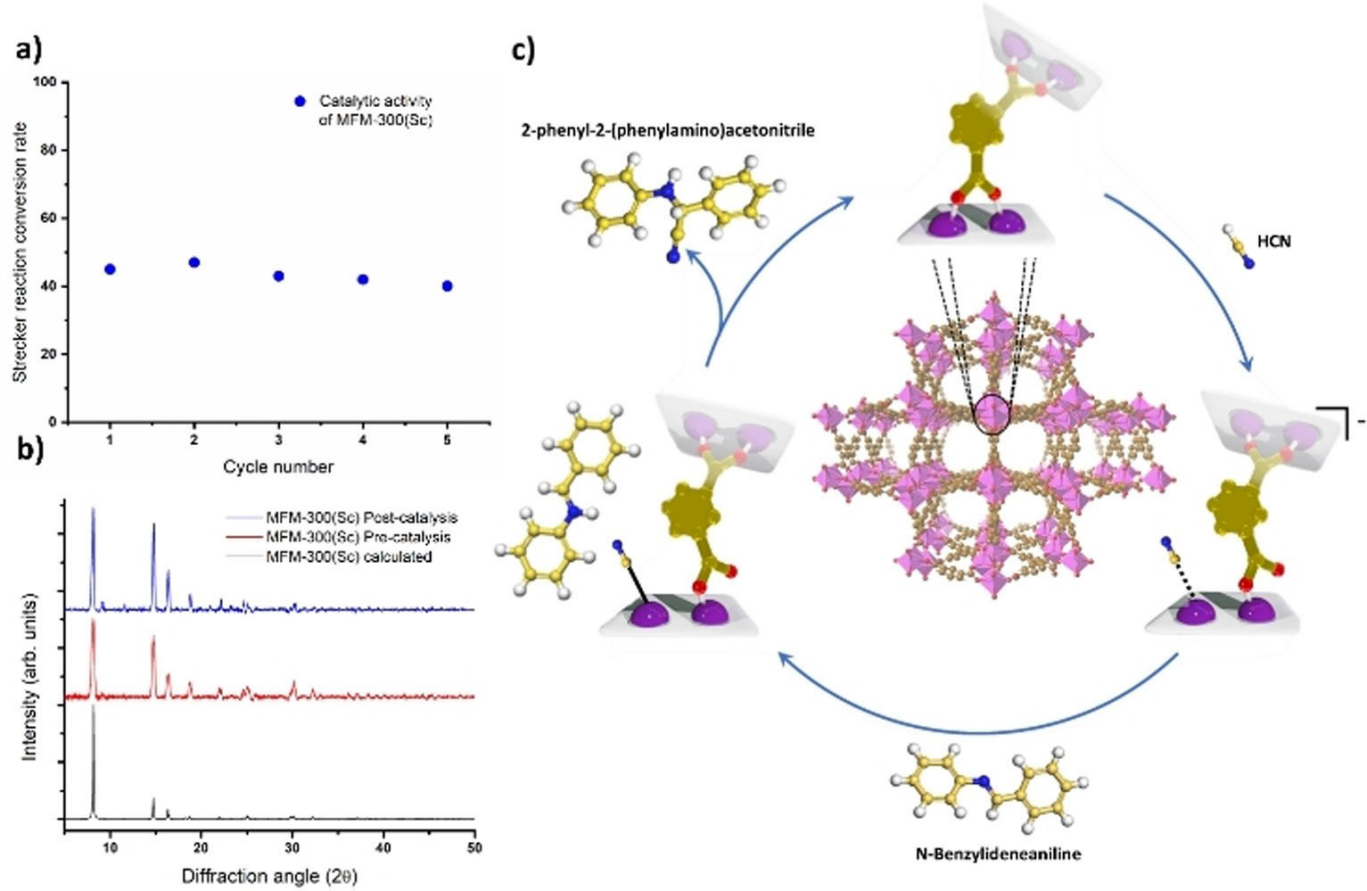
a) Catalytic activity of MFM‐300(Sc) over 5 cycles, b) PXRD data of the MOF sample before and after 5 cycles catalysis reaction, c) Illustration of the catalytic mechanism for the production of 2‐phenyl‐2‐(phenylamino)acetonitrile driven by the metal‐hemilabile linker dynamics in MFM‐300(Sc) (C, gold; H, white; N, blue; O, red; Sc, pink.

Intuitively, OMS induced by the presence of missing linkers in the MOF framework could be the origin of this catalytic activity.^[34]^ Thus, five different batches of MFM‐300(Sc) were produced containing different concentrations of missing linkers ranging from 5 % to 19 % as determined by NMR experiments (see Table 1). Interestingly, the conversion rate remains almost the same whatever the defective linker concentration was tested. This observation supports that the Stretcher reaction is not driven by OMS created by the missing linkers and indeed suggests that the metal‐ligand bonding dynamics we previously revealed upon NH_3_ adsorption, might be at the origin of the catalytic activity of this Sc^III^‐based MOF. Importantly, MFM‐300(Sc) exhibited to retain its catalytic performance over 5 cycles (Figure 2a). PXRD and NMR experiments confirmed the retention of the crystallinity (Figure 2b) and an almost constant fraction of missing linkers on five independent samples where each sample was cycled 5 times (Figure S7). This highlights a fully reversible catalytic mechanism without any alteration of the catalyst and without losing its catalytic performance.

**Table 1:** Fraction of missing linkers (experimentally calculated linkers/ theoretical linkers *100) in five different batches of MFM‐300(Sc) determined by NMR spectroscopy and their corresponding conversion rate following the Strecker reaction illustrated in Scheme 1.

Trial	1	2	3	4	5
Conversion %	45	42	50	51	47
Missing Linker %	8	19	5	14	17

Another possibility to analyze is Sc‐solvent species which could be located at the surface of the MOF and thus, these may be also responsible for the catalytic activity of MFM‐300(Sc). Indeed, some Sc^III^ species, such as scandium(III) triflate hydrate^[35]^ or scandium(III) chloride, can be formed due to the use of HCl for the synthesis of this MOF or post‐synthesis treatments.[^25^, ^36^] In addition, if these species are present at the surface of this MOF, these can also strongly interact in the pores with the polar hydroxyl groups (μ_2_‐OH) inside the pores of MFM‐300(Sc).^[37]^ In order to elucidate this hypothesis, we first performed EDX analyses different MFM‐300(Sc) samples and it was possible to ruled out the presence of such species (see Figure S5). Further, if such large complexes would be trapped inside the pores of this MOF, the BET area and the pore size distribution of MFM‐300(Sc) should be drastically reduced. Thus, a N_2_ adsorption isotherm on one of this investigated samples was collected at 77 K, affording a calculated BET area of approximately 1385 m^2^ g^−1^ with a total pore volume of 0.56 cm^3^ g^−1^ (see Figure S4). These values are both in excellent agreement with the theoretical values calculated from the crystal structure using a geometric method (1390 m^2^ g^−1^ and 0.58 cm^3^ g^−1^ respectively).^[38]^ Moreover, the pore size distribution plot confirms an homogeneous pore size distribution of approximately 7.5 Å, consistent with the values determined from the single‐crystal X‐ray structures of 8.1 Å.^[39]^ All together, this data supports the absence of scandium (III) complexes both at the surface of MFM‐300(Sc) and within its pores, which clearly discards their involvement in the catalytic reaction.

Amorphous MOFs have also been reported as responsible for potentially active materials for different catalytic reactions.^[40]^ Thus, to eliminate the hypothesis that the presence of amorphous impurities may influence the catalytic activity of MFM‐300(Sc), Scanning Electron Microscopy (SEM) and Energy Dispersive X‐Ray (EDX) were performed, in addition to the collection of PXRD patterns, which already evidenced high crystallinity of the MFM‐300(Sc) sample. SEM analysis demonstrated the presence of only capsule‐shaped MOF crystals, while EDX mapping of pre‐ and post‐catalysis samples revealed a homogeneous distribution of scandium atoms for MFM‐300(Sc) which confirms the retention of the crystalline structure.

Finally, the hydroxyl groups within the pores of MFM‐300(Sc) have previously exhibited activity for acid‐catalyzed Friedel–Craft alkylation of different aromatic molecules.^[41]^ The acidic strength of these hydroxyl groups varies depending to the metals that they are coordinated to. However, this acidity is generally weak. To eliminate the possibility of these groups taking part on the catalytic activity for the Strecker reaction, we performed the reaction using D_2_O. By performing this reaction with D_2_O, we predicted that the D_2_O will react with TMSCN to generate DCN and ultimately D^+^. Thereby the amine “NH” site in the product should be predominantly deuterated. ^1^H NMR (Scheme S1, Figure S9 and Figure S10) reveals the absence of a peak at 4.27 ppm from the NH. Furthermore, as demonstrated by Schröder and co‐workers, a visible exchange in the OH band (from around ν(OH): 3692 to ν(OD): 2720 cm^−1^) should be observed if an exchange of H/D occurs within MFM‐300(Sc).^[39]^ Hence, infrared (IR) spectroscopy was performed on the material after the catalysis and confirmed an unaltered shift for the OH band This confirmed that no OD species were formed. Thus, the only source of D^+^ is the one ultimately formed via the interaction between D_2_O and TMSCN.

**Figure 3:**
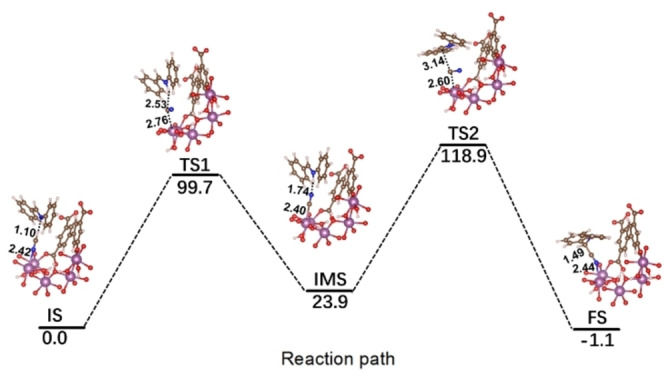
Potential energy profile for the reaction mechanism between N‐benzylideneaniline and HCN within MFM‐300(Sc) initiated by the adsorption of HCN which implies the formation of a Sc−CN complex in the first step of the reaction The initial state is taken as the references of the potential energy. Colors code: carbon (brown), hydrogen (white), nitrogen (blue), oxygen (red) and scandium (purple). The energies and distances are reported in kJ mol^−1^ and in Å, respectively.

This comprehensive experimental characterization is robust enough to support the hypothesis that the Sc^III^ OMS are created by the metal‐linker bond dynamics, as previously revealed by our computational simulations on the NH_3_ adsorption‐desorption for MFM‐300(Sc).^[21]^ Thus, periodic DFT calculations were further performed to unravel the catalytic mechanism of the Strecker reaction over these Sc^III^ OMS. We first computationally assess the potential formation of Sc^III^ OMS in the presence of HCN as summarized in Figure S8 following the same approach than in our previous paper on NH_3_ adsorption^[24]^ (see Supporting Information for methodology). The corresponding activation energy of 68.3 kJ mol^−1^ was found to be moderate and potentially overcome over the reaction. Therefore, the formation of Sc^III^ OMS can be expected under our experimental working conditions. For all calculations on the reaction mentioned above, HCN instead of TMSCN was considered as a model molecule‐reactant that reacts with *N*‐benzylideneaniline owing to its facile hydrolysis under the experimental conditions with wet chloroform‐d. We first confirmed that the reaction without the presence of Sc^III^ species is hardly feasible at room temperature since it is associated with a very high energy barrier (163.0 kJ mol^−1^) and an exothermic reaction energy (−8.0 kJ mol^−1^), as illustrated in Figure S12. We further explored a standard Strecker mechanism with an initial step implying a bond formation between C(HCN) and the Sc^III^ OMS (Initial State: IS‐Figure 3) (note the length of a typical Sc−C bond is around 2.2–2.3 Å.^[42]^ The potential energy profile and the corresponding initial state (IS), transition states (TS), intermediate state (IMS) and final state (FS) encountered along this minimum energy pathway are reported in Figure 3. The first step of the reaction proceeds via a proton transfer from HCN to the N(imine) as well as a flipping of the cyanide species at the first transition state (TS1) and the remaining −CN group binds stronger to the open Sc^III^ site, as it was revealed by the shortening of the Sc−C(CN) bond length to 2.40 Å in an IMS associated with an activation barrier of 99.7 kJ mol^−1^. The second step of the reaction proceeds via a second transition state (TS2) corresponding to the flip of −CN to −NC group to initialize a nucleophilic attack to the iminum intermediate species formed in the first step with a resulting activation barrier of 95.0 kJ mol^−1^. The overall reaction energy (118.9 kJ mol^−1^) is lower than the than the value simulated for the reaction in the absence of the catalyst (163 kJ mol^−1^) (Figure S12). This predicted trend is in line with the difference in terms of yields experimentally observed for the MFM‐300(Sc)‐catalyzed and uncatalyzed situations (45 % versus 0.05 %). Remarkably, the reaction energy is negative (−1.1 kJ mol^−1^), indicating that such process is not only kinetically but also thermodynamically feasible. Note that the trend remains the same if it is considered an unconventional Strecker mechanism via the formation of a non‐standard Sc−N(imine) bond as previously proposed by a combined experimental and theoretical investigation on scandium isocyanide molecules^[43]^ (see Figure S13–15 and Supporting Information for more details).

## Conclusion

In summary, the prototypical MOF‐type MFM‐300(Sc) with non‐permanent open metal sites was demonstrated to act as a catalyst owing to a metal‐hemilabile linker bond dynamics of the MOF framework that enables to switch on/switch off open metal sites. As a proof‐of‐concept, the MOF‐catalyzed formation of 2‐phenyl‐2‐(phenylamino)acetonitrile from the reaction between N‐Benzylideneaniline and TMSCN via the Strecker hydrocyanation reaction was experimentally demonstrated. The catalytic activity of MFM‐300(Sc) was evidenced to be fully reversible with its conversion performance and structure integrity maintained over multiple cycles. Quantum‐calculations confirmed the pivotal role played by the non‐permanent Sc‐open metal sites of the MOF in the catalytic reaction mechanism. These key findings pave the way towards new horizons to the design of heterogeneous MOF catalysts easily regenerable while maintaining high‐level performance over cycling.

## Conflict of interest

The authors declare no conflict of interest.

1

## Supporting information

As a service to our authors and readers, this journal provides supporting information supplied by the authors. Such materials are peer reviewed and may be re‐organized for online delivery, but are not copy‐edited or typeset. Technical support issues arising from supporting information (other than missing files) should be addressed to the authors.

Supporting Information

## Data Availability

The data that support the findings of this study are available from the corresponding author upon reasonable request.
