# Supplementary material for: Switchable Metal Sites in Metal–Organic Framework MFM‐300(Sc): Lewis Acid Catalysis Driven by Metal–Hemilabile Linker Bond Dynamics
Source: Angew Chem Int Ed Engl. 2022 Oct 25;61(48):e202210857. doi: 10.1002/anie.202210857 (PMC9828200; doi:10.1002/anie.202210857)
Supplement: Supplementary file 1 — Supporting Information [file ANIE-61-0-s001.pdf]

Angewandte Chemie
Eine Zeitschrift der Gesellschaft Deutscher Chemiker

Supporting Information

Switchable Metal Sites in Metal–Organic Framework MFM-300(Sc): Lewis Acid Catalysis Driven by Metal–Hemilabile Linker Bond Dynamics

R. A. Peralta, P. Lyu, A. López-Olvera, J. L. Obeso, C. Leyva, N. C. Jeong, I. A. Ibarra*, G. Maurin**

Table of Contents

Experimental section

S1. Experimental methods

S2. Characterization of MFM-300(Sc)

S3. Catalysis measurements

S4. DFT calculations

S5. References

Experimental section

S1. Methods

Materials. The following reagents were purchased from commercial vendors and used as received. Benzyl alcohol (99 %, Alfa Aesar), aniline (≥ 99 %, Alfa Aesar), NaOH (≥ 98 %, pellets (anhydrous), Sigma Aldrich), trimethylsilyl cyanide (98 %, Sigma Aldrich), scandium triflate (99 %, Sigma Aldrich), Biphenyl-3,3′,5,5′-tetracarboxylic acid (H_4BPTC) (≥ 99.7 %, Sigma Aldrich), chloroform (≥ 99 %, ≥ 0.056 % of H_2O, Sigma Aldrich), chloroform-d (99.8 atom % D, ≥ 0.056 % of H_2O, Sigma Aldrich), DMSO-d6 (99.9 atom % D, Sigma Aldrich), deuterium chloride solution (35 wt. % in D_2O, ≥ 99 atom % D, Sigma Aldrich), dichloromethane (≥99.8 %, Sigma Aldrich), HCl (36.5 wt. % in H_2O, Sigma Aldrich), THF (anhydrous, ≥ 99.9 %, Sigma Aldrich), DMF (≥ 99.8 %, Sigma Aldrich) and petroleum ether (puriss. p.a., high boiling, bp 60-80 °C, Sigma Aldrich) were purchased from commercial vendors and used as received. The chloroform-d was dried over literature procedures and degassed with Ar prior to use.

MFM-300(Sc) Synthesis and characterization. Following a previously reported procedure,[1] scandium triflate (0.030g, 0.061 mmol) and H_4BPTC (0.010 g, 0.030 mmol) were mixed in THF (4.0 ml), DMF (3.0 ml), water (1.0 ml) and HCl (36.5 %, 2 drops). The resultant slurry mixture was stirred until complete dissolution occurred. The solution was then placed in a pressure tube and heated in an oil bath to 75 °C for 72 h. The tube was cooled down to room temperature at a rate of 0.1 °C/min, and the colorless crystalline product was separated by filtration, washed with DMF (5.00 ml) and dried in air. Samples were handled under standard Schlenk techniques unless otherwise stated. N-Benzylideneaniline was synthesized and purified as previously reported.[2]

Powder X-ray diffraction (PXRD) data were collected on a Bruker Advanced D4 diffractometer using Cu Kα radiation (λ = 1.5456 Å , 40 kW/ 40mA, 2θ = 5 – 50ϕ, phi rotation = 20 rotation/min, at 1 sec exposure per step with 5001 steps and using 0.5 mm glass capillaries). NMR spectra were recorded on Varian Gemini 400 MHz spectrometers at 25 °C using a 5 mm probe.

Powder X-ray diffraction (PXRD) patterns were recorded with a Bruker Advance II diffractometer equipped with a θ/2θ Bragg-Brentano geometry and Ni-filtered CuKα radiation ($K\alpha1$ = 1.5406 Å, $K\alpha2$ = 1.5444 Å, $K\alpha1/K\alpha2$ = 0.5). The tube voltage and current were 40 kV and 40 mA, respectively. Samples for PXRD were prepared by placing a thin layer of the appropriate material on a zero-background silicon crystal plate.

Nitrogen adsorption isotherms were measured by a volumetric method using a Micromeritics ASAP 2020 gas sorption analyzer. The sample mass was 65.0 mg. Free space correction measurements were performed using ultra-high purity He gas (UHP grade 5, 99.999% pure). Nitrogen isotherms were measured using UHP grade Nitrogen. All nitrogen analyses were performed using a liquid nitrogen bath at 77 K. Oil-free vacuum pumps were used to prevent contamination of sample or feed gases.

NMR spectra were recorded using an AVANCE III HD FT-NMR spectrometer (Bruker, 400 MHz for ^{1}H). The ^{1}H and ^{13}C chemical shifts were referenced to the residual proton resonance of the solvent.

Infrared (IR) spectroscopy were obtained using an FT-IRMicroscope (Thermo Scientific, Nicolet Continuum).

Scanning electron microscopy (SEM) images were obtained from an FE-SEM (Hitachi S-4800) operated at an acceleration voltage of 3 kV, after samples were coated by Pt alloys with a thickness of approximately 3 nm.

S2. Characterization of MFM-300(Sc)

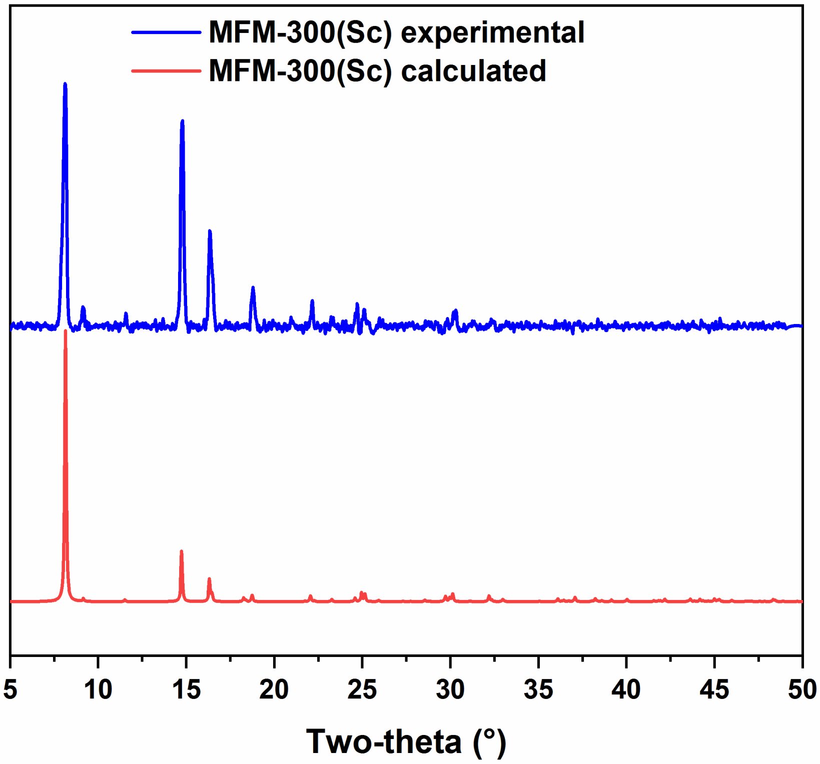

Figure S1: Experimental (blue) and simulated (red) powder X-ray diffraction pattern of MFM-300 (Sc).

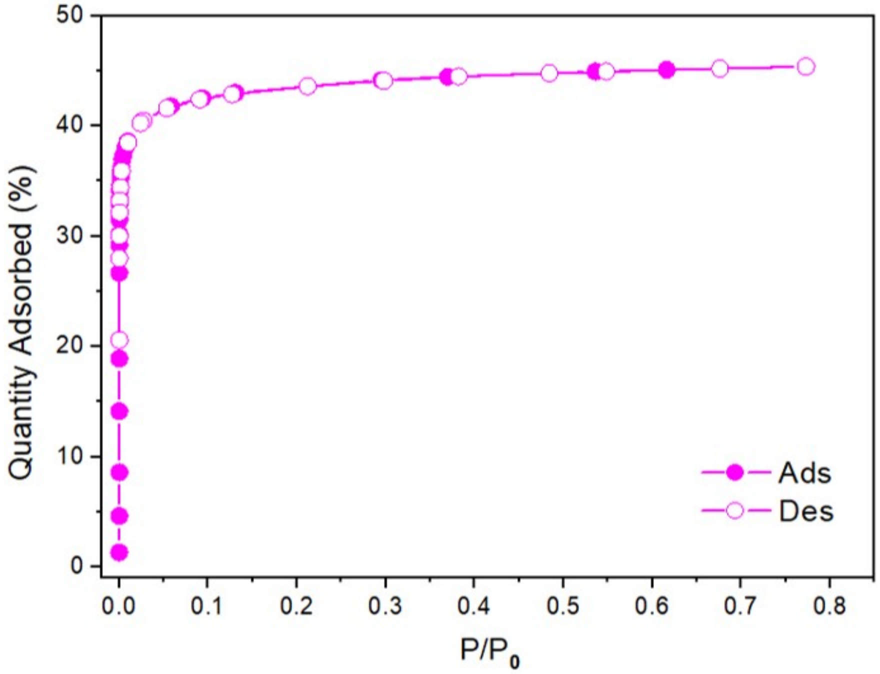

Figure S2: N_2 adsorption/desorption isotherms at 77 K of the as-synthesized MFM-300(Sc).

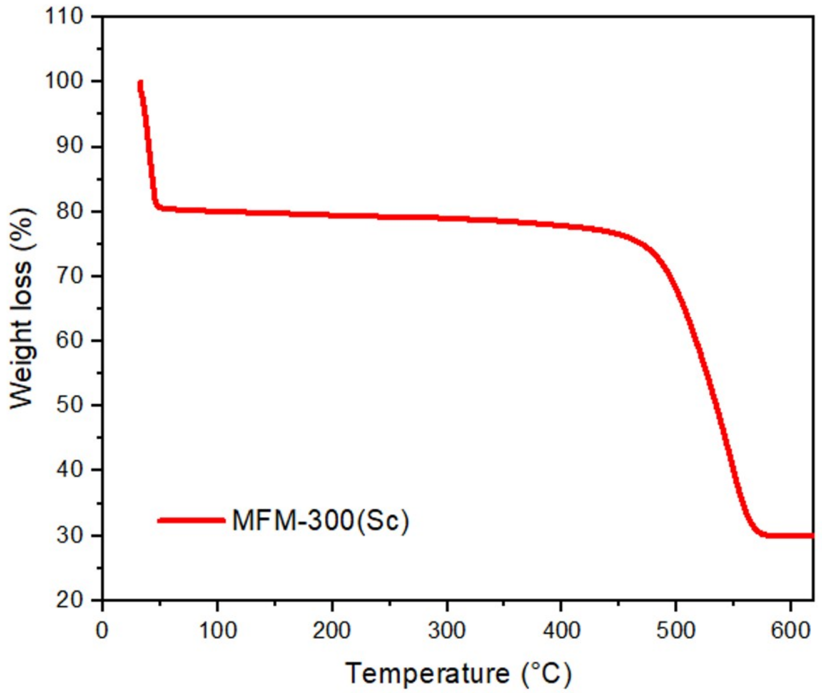

Figure S3: Thermogravimetric analysis of the as-synthesized MFM-300(Sc) material.

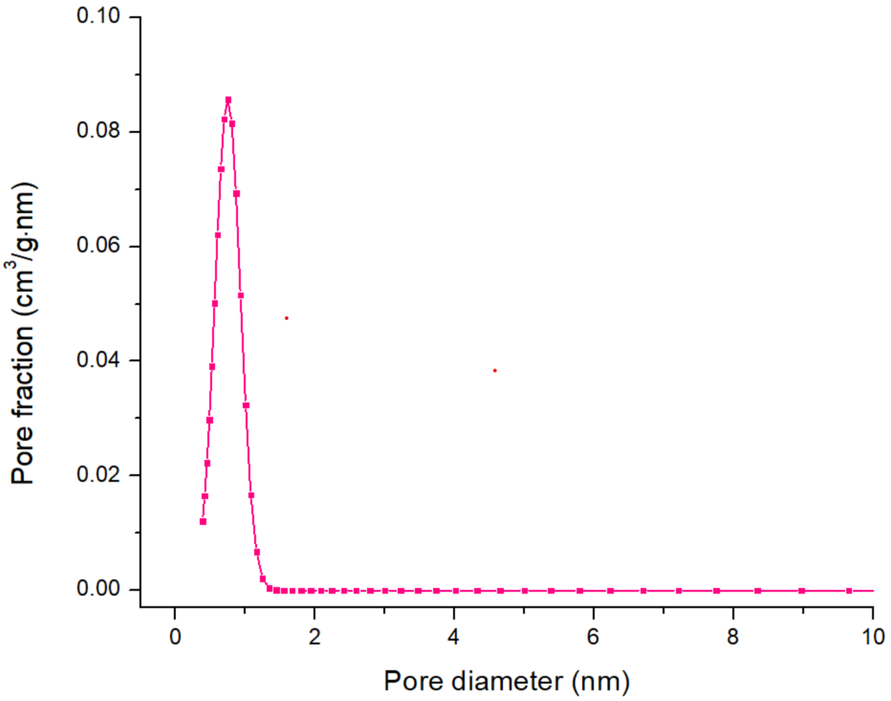

Figure S4. DFT pore size distributions of MFM-300 (Sc)

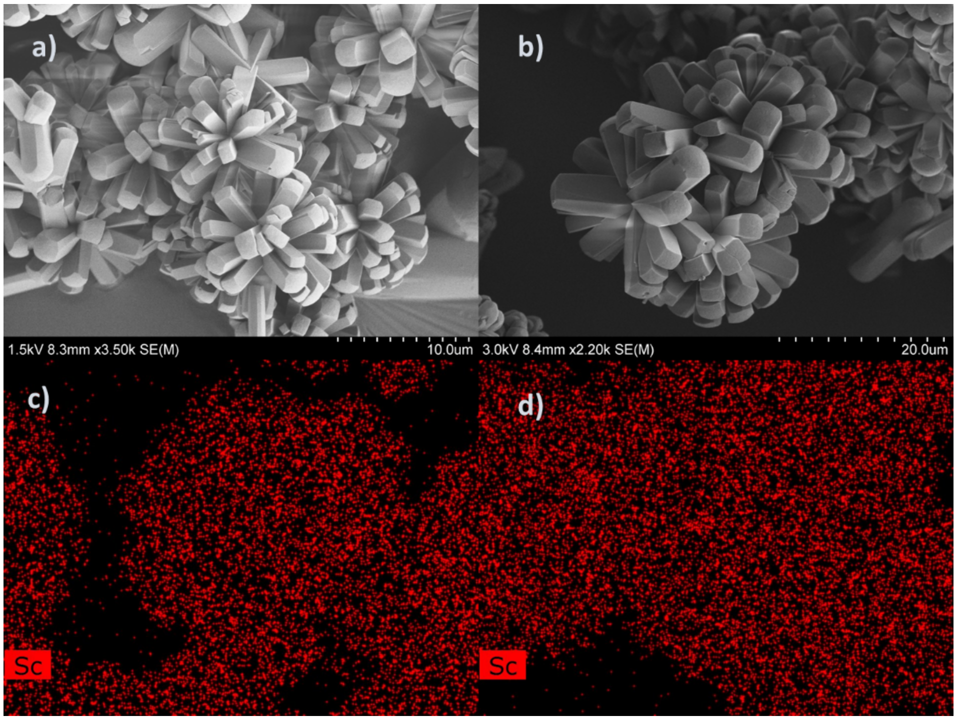

Figure S5. SEM images with EDX analysis. SEM images of MFM-300 (Sc) a) pre- catalysis and b) post-catalysis. c) SEM EDX mapping MFM-300 (Sc) a) pre-catalysis d) post-catalysis.

S3. Catalysis measurements

Catalytic synthesis of 2-phenyl-2-(phenylamino)acetonitrile small-scale. The synthesized MFM-300(Sc) (approximately 22mg) were washed with DMF (5 x 4mL) and subsequently chloroform (5 x 4mL), the chloroform was degassed with Ar after each exchange, and the crystals were allowed to soak 1 h between exchanges. The crystals were activated to remove the pore-filling solvent at 60°C for 16 hours and stored inside a glove box. Further, MFM-300(Sc) sample (2 mg, 0.0023 mmol) was placed in a 4 mL glass vial, and $CDCl_3$ (0.07 mol% of H_2O, 1.5 ml), N-Benzylideneaniline (31.5 mg, 0.173 mmol), and trimethylsilyl cyanide (86.21 mg, 0.869 mmol) were added and the vial sealed at RT for 96 h. This process was repeated 5 more times to obtain the results in Table 1. The extent of conversion was calculated by comparing the reduction in the integral of the alkene CH resonance of N-Benzylideneaniline at 8.46 ppm and the appearance of a new secondary amine NH resonance from 2-phenyl-2-(phenylamino)acetonitrile at 4.27 ppm.

Large-scale generation and purification of 2-phenyl-2-(phenylamino)acetonitrile. The synthesized MFM-300(Sc) (approximately 22mg) was washed with DMF (5 x 4mL) and subsequently chloroform (5 x 4mL), the chloroform was degassed with Ar after each exchange, and the crystals were allowed to soak 1 h between exchanges. Under argon, N-Benzylideneaniline (350 mg, 1.93 mmol) and trimethylsilyl cyanide (957.97 mg, 9.65 mmol) were added to the MFM-300(Sc) crystals, and the vial was sealed at RT for 96 h. The MOF crystals were isolated *via* filtration and washed with chloroform (0.07 mol% H_2O, 30 ml). The chloroform was removed under reduced pressure. ^{1}H NMR indicated the formation of the product, which purified by silica gel column chromatography (dichloromethane: petroleum ether (2:1)) to afford pure 2-phenyl-2-(phenylamino)acetonitrile as a white solid (140 mg, 35% Yield). The reaction conditions have not been optimized. ^{1}H NMR (400 MHz, $CDCl_3$); δ (ppm): 7.5-7.32 (m, 2H) 7.37-7.29 (m, 3H) 7.3-7.27 (m, 2H), 6.89-6.79 (m, 3H), 5.41 (s, CH), 4.27 (s, NH). ^{13}C NMR (101 MHz, $CDCl_3$); δ (ppm): 145.3, 134.6, 128.7, 128.2, 127.4, 120.5, 118.7, 114.2, 52.1.

Defect concentration in MFM-300(Sc). The synthesized MFM-300(Sc) (approximately 22mg) were washed with DMF (5 X 4mL) and subsequently chloroform (5 x 4mL), the chloroform was degassed with Ar after each exchange, and the crystals were allowed to soak 1 h between exchanges. The crystals were activated to remove the pore-filling solvent at 60°C for 16 hours and stored inside a glove box. Further, MFM-300(Sc) sample (7 mg) was placed in an NMR tube were with 2 μL of THF, and the crystals dissolved in DMSO-d_6 (deuterated dimethyl sulfoxide, 580 μL) and 20% DCl in D_2O (20 μL) for ^{1}H NMR analysis. This process was repeated 5 more times to obtain the data in Table 1. Finally, 5 different samples were recycled and digested in every cycle to obtain the Figure S7. The defects in MCM-300(Sc) were calculated by comparing the integral of the four aromatic CH of Biphenyl-3,3′,5,5′-tetracarboxylic acid 8.27-8.44 ppm and the integral of the two CH_2 of THF at 3.78 ppm and 1.86 ppm. (Figure S6).

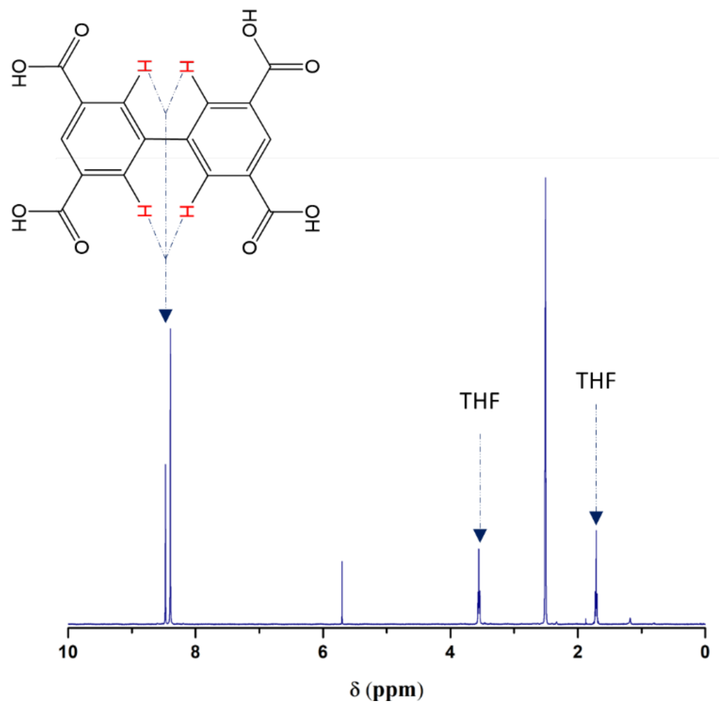

Figure S6. Comparison of the ligand in MFM-300 and THF (as reference), methodology used to calculate the number of missing linkers.

Homogenous Strecker reaction with Sc(III) triflate. In a 4ml vial, Sc(III) triflate (0.17 mmol, 10 mol%), N-Benzylideneaniline (31.5 mg, 0.173 mmol), and trimethylsilyl cyanide (957.97 mg, 9.65 mmol) were added in 1.5 ml of $CDCl_3$. Successively, the vial was stirred and closed under Ar at room temperature for 20 hours to yield 2-phenyl-2-(phenylamino)acetonitrile. ^{1}H NMR (400 MHz, $CDCl_3$); δ (ppm): 7.5-7.32 (m, 2H) 7.37-7.29 (m, 3H) 7.3-7.27 (m, 2H), 6.89-6.79 (m, 3H), 5.41 (s, CH) 4.27 (s, NH).

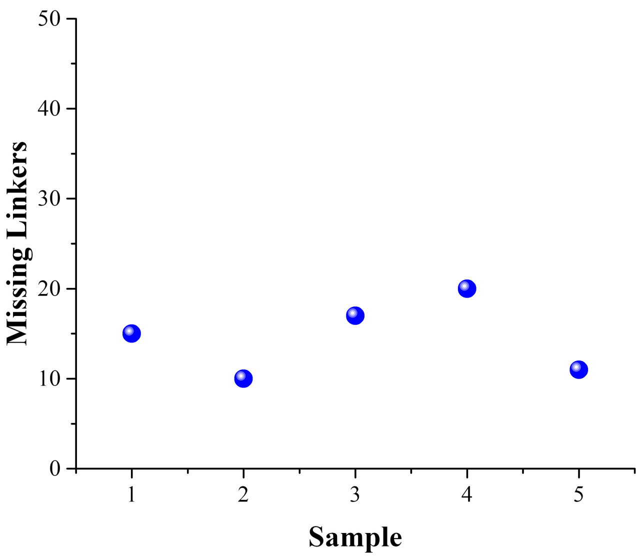

Figure S7. Missing linker concentration determined in five independent samples that were five times cycled.

Effect of water on the Strecker reaction catalyzed by MFM-300(Sc). The synthesized MFM-300(Sc) (approximately 22mg) crystals were washed with DMF (5 X 4mL) and subsequently with chloroform (5 x 4mL). The chloroform was outgassed with Ar after each exchange and the crystals were allowed to soak 1 h between exchanges. The crystals were activated to remove the pore-filling solvent at 60 °C for 16 hours and stored inside a glove box. Further, MFM-300(Sc) sample (2 mg, 0.0023 mmol) was placed in a 4 mL glass vial, and $CDCl_3$ (0.07 mol% of H_2O, 1.5 ml), N-Benzylideneaniline (31.5 mg, 0.173 mmol), and trimethylsilyl cyanide (86.21 mg, 0.869 mmol) were added and the vial sealed at RT for 96 h. This process was repeated 4 more times with different amounts of water (0, 0.01, 0.02, 0.03, and 0.05 % of water in 1.5 ml of $CDCl_3$) to obtain the results summarized in Figure S8. The extent of conversion was calculated by comparing the reduction in the integral of the alkene CH resonance of N-Benzylideneaniline at 8.46 ppm and the appearance of a new secondary amine NH resonance from 2-phenyl-2-(phenylamino)acetonitrile at 4.27 ppm.

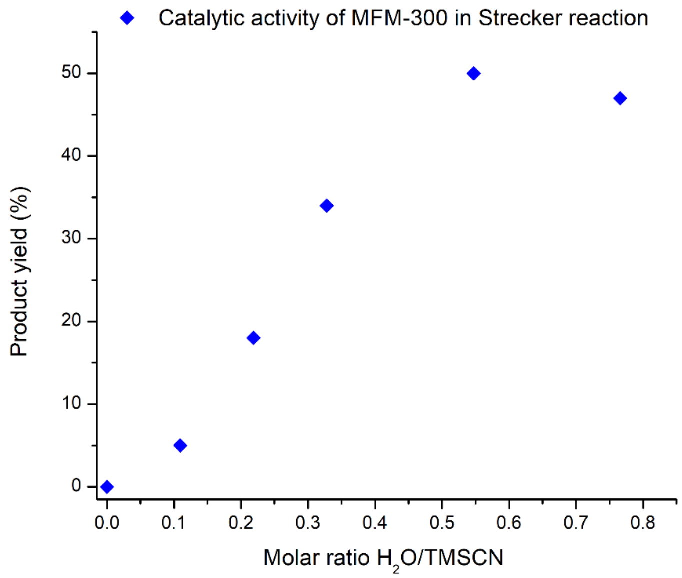

Figure S8. Effect of water in the reaction (molar ratio of H_2O/TMSCN) compared to the product yield of 2-phenyl-2-(phenylamino)acetonitrile.

Catalytic generation of deuterated 2-phenyl-2-(phenylamino)acetonitrile. The synthesized MFM-300(Sc) (approximately 22mg) were washed with DMF (5 x 4mL) and subsequently chloroform (5 x 4mL), the chloroform was degassed with Ar after each exchange, and the crystals were allowed to soak 1 h between exchanges. The crystals were activated to remove the pore-filling solvent at 60°C for 16 hours and stored inside a glove box. Further, MFM-300(Sc) sample (2 mg, 0.0023 mmol) was placed in a 4 mL glass vial, and $CDCl_3$ (0.07 mol% of D_2O, 1.5 ml), N-Benzylideneaniline (31.5 mg, 0.173 mmol), and trimethylsilyl cyanide (86.21 mg, 0.869 mmol) were added and the vial sealed at RT for 96 h. The extent of conversion was about 39 %, calculated by comparing the reduction in the integral of the alkene CH resonance of N-Benzylideneaniline at 8.46 ppm and the appearance of a new alkane CH resonance from 2-phenyl-2-(phenylamino)acetonitrile at 5.41 ppm. (Figure S9).

Scheme S1. Strecker reaction catalyzed by MFM-300(Sc) in presence of D_2O.

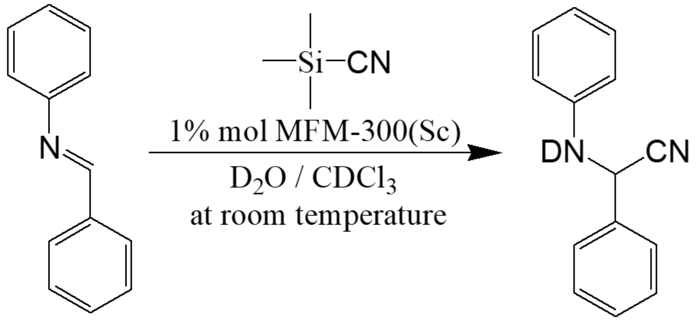

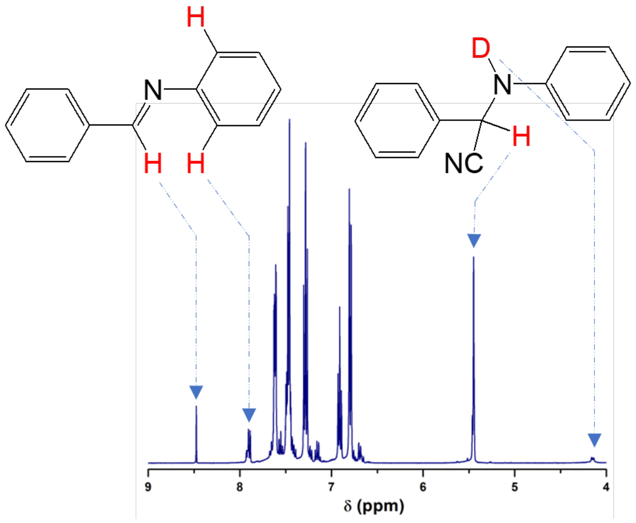

Figure S9. NMR spectra of post-catalysis utilizing D_2O as a proton source.

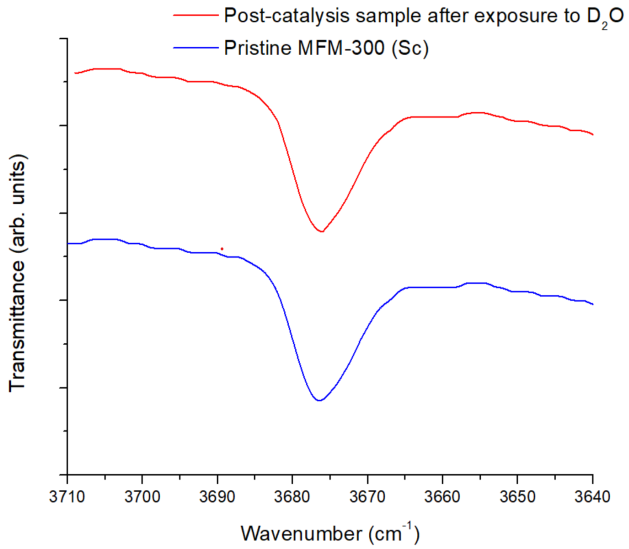

Figure S10. IR spectra of MFM-300 (Sc) pre-and post-catalysis in the μ-OH stretching range which demonstrates an unaltered shift of the band.

S4. Computational details

Periodic DFT calculations were performed using the projector augmented wave (PAW) method with electron exchange–correlations described by the Perdew-Burke-Ernzerhof (PBE) functional within the generalized gradient approximation (GGA) scheme[3][45] as implemented in the Vienna Ab Initio Simulation Package (VASP).[4-6][46-48] The convergence criteria for the forces and energy during the geometry optimizations were fixed to 0.01 eV/Å (0.03 eV/Å for transition state searching) and 10-5 eV, respectively. The DFT-D3 correction was used to account for the dispersion contribution.[7][49] The climbing image nudged elastic band (CI-NEB) method[8][50] within the Transition State Tools for VASP (VTST) module was employed to identify the transition states. The Brillouin zone was sampled at the gamma point. The simulation cell made of 1 x 1 x 2 unit cells of MFM-300(Sc) was considered to avoid interactions between the relatively long N-benzylideneaniline molecule (length ~11.5 Å) and its image length (see Fig. S11). Our previously reported DFT optimized crystal structure of MFM-300(Sc) containing an open Sc site resulting from the Sc-O bond breaking and the dangling carboxylate group interacts with μ-OH groups, and leaving the 5-coordinated Sc as an catalytically active site.

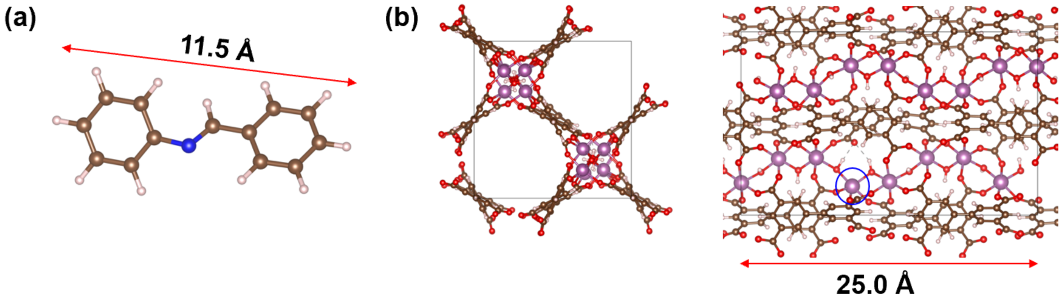

Figure S11: (a) Optimized structure of N-Benzylideneaniline molecule; (b) Views from c (left) and b (right) directions of the optimized double cell of MFM-300(Sc) integrating an open metal Sc site (blue circle). Colors code: carbon (brown), hydrogen (white), nitrogen (blue), oxygen (red) and scandium (purple).

DFT calculations

Trimethylsilyl cyanide (TMS-CN) can be easily hydrolyzed to trimethylsilanol and hydrogen cyanide (HCN) particularly under humid air. Therefore, our DFT exploration assumed that the Strecker reaction proceeds in the MFM-300(Sc) pores with HCN and N-benzylideneaniline as reactants in the presence of H_2O since it is highly improbable that the two bulky molecules TMS-CN and N-benzylideneaniline are co-hosted by the wine track MOF channel of only 8.1 Å.

As an initial step, the mechanism of this model reaction was investigated without any catalysts. The corresponding DFT-calculated potential energy profile reported in Figure S11 shows that at the initial state (IS), HCN interacts with the N-benzylideneaniline molecule via the formation of an H—N hydrogen bond of 2.27 Å. The reaction then proceeds via a proton transfer from HCN to the Nitrogen atom of N-benzylideneaniline leading to the formation of a -NH- function at the transition state (TS) followed by the creation of a -CH(CN)- group at the final state (FS). Although this reaction was found to be exothermic (reaction energy of -8.0 kJ mol^{-1}), its calculated energy barrier is rather high (163 kJ mol^{-1}). This emphasizes that that this reaction cannot be achieved at room temperature without the use of a catalyst.

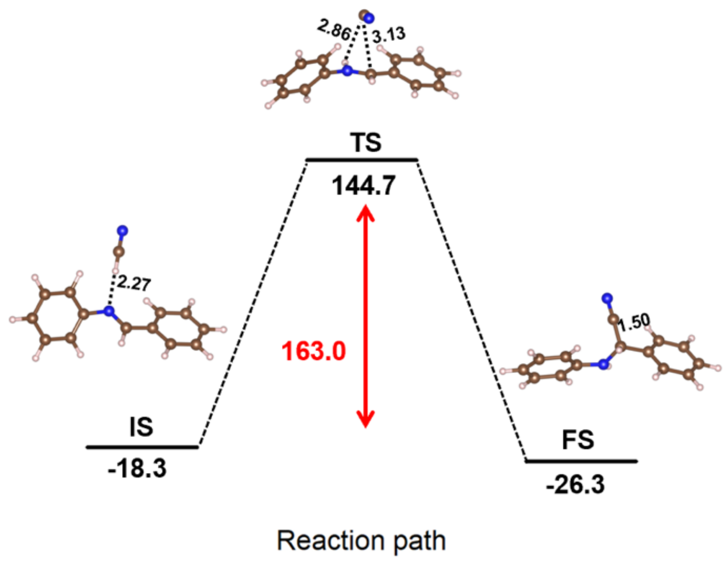

Figure S12: DFT-potential energy profile for the reaction mechanism between N-benzylideneaniline and HCN without the use of a catalyst. The isolated molecules are taken as references of the potential energy. Colors code: carbon (brown), hydrogen (white) and nitrogen (blue). The energies are in kJ mol^{-1} and the distances are in Å, respectively.

We further investigated the same reaction catalyzed by MFM-300(Sc) with a mechanism initiated by the formation of Sc- N(imine) interactions as typically considered for Strecker reaction catalyzed by scandium triflate.[3] Figure S12 shows that the DFT-optimized adsorption configuration (ADS) of N-benzylideneaniline in the pore of MFM-300(Sc) is associated with a rather weak Sc-N¬(imine) interaction (separating distance of 4.03 Å) mostly due to the bulky configuration of the imine molecule. This holds also true at the IS of the reaction once HCN is added with a Sc-N¬(imine) separating distance of 4.50 Å. The corresponding reaction mechanism implies an energy barrier (172.0 kJ mol^{-1}) as high as the value (163.0 kJ mol^{-1}) obtained for the un-catalyzed scenario (Figure S12), excluding any catalytic role of MFM-300(Sc) with the consideration of this standard reaction scheme.

Methodology for the formation of dynamic open metal site

The DFT calculations have been performed using the projected augmented wave (PAW)[3] formulism within the non-local vdw-DF2[6] as implemented in Vienna ab initio simulation package (VASP).[4-6] The convergence criterion of 0.01eV/Å for the force was adapted for geometry optimizations (0.02eV/Å for transition state searching), while the criterion for self-consistent field (SCF) is 10^{-5} eV. To figure out possible product of NH_3 reacts with MFM-300(Sc), the reaction

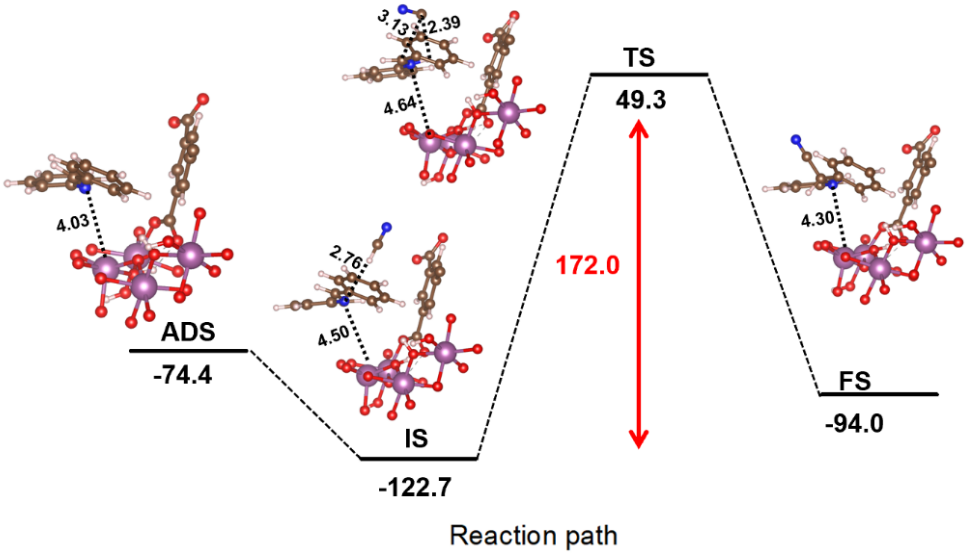

Figure S13: Potential energy profile for the reaction mechanism between N-benzylideneaniline and HCN in the MFM-300(Sc) initiated by the adsorption of N-benzylideneaniline. The isolated molecules are taken as the references of the potential energy. Colors code: carbon (brown), hydrogen (white), nitrogen (blue), oxygen (red) and scandium (purple). The energies are in kJ mol^{-1} and the distances are in Å, respectively.

paths were searched with the climbing image nudged elastic band method (CI-NEB)[8] as implemented in the Transition State Tools for VASP (VTST)[9] and verified with frequency calculations. All DFT calculations are performed at gamma point, while the cutoff energy of 900eV for the plane-wave basis set has been consistently used. The lattice parameters optimized for the unit cell of MFM-300(Sc) are a=b=15.47 Å, c=12.52 Å, α=β=γ=90º.

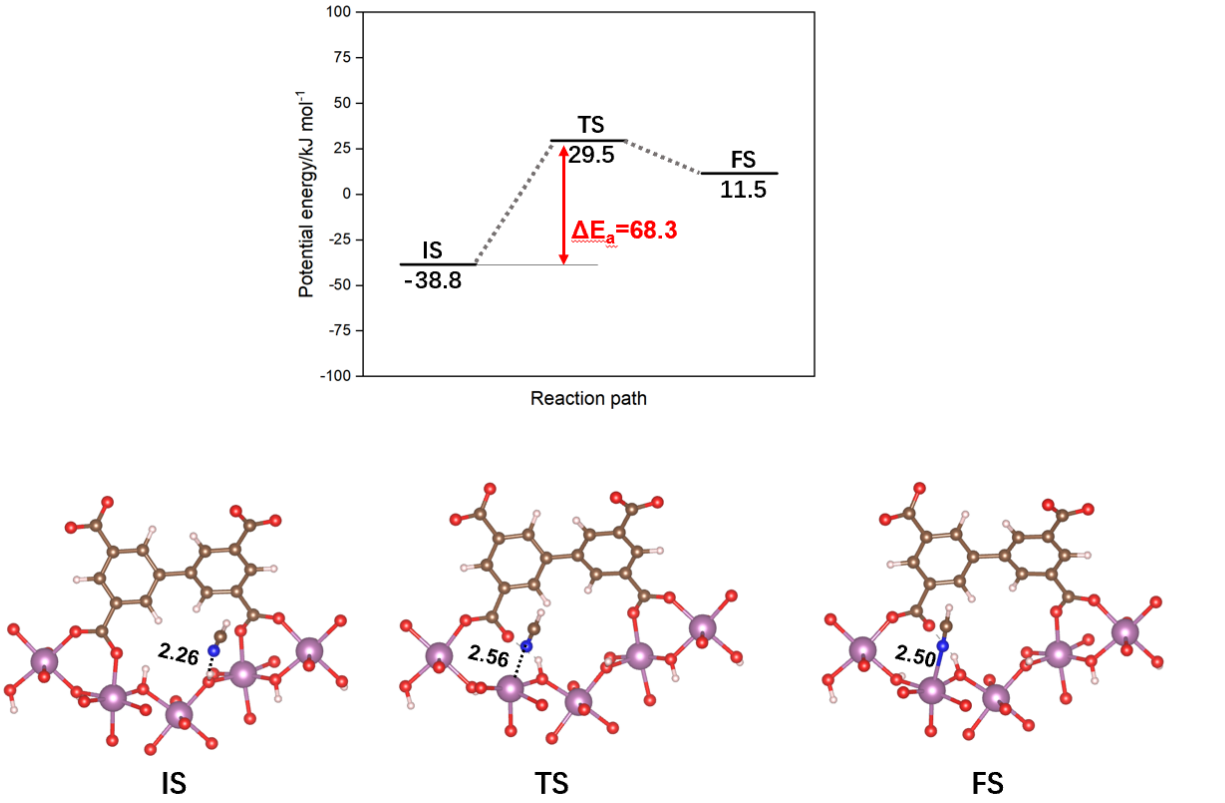

Figure S14: Potential energy profile for the formation mechanism of the dynamic open Sc site. The empty MOF structure and isolated HCN molecule are taken as the references of the potential energy. Colors code: carbon (brown), hydrogen (white), nitrogen (blue), oxygen (red) and scandium (purple). The energies and distances are reported in kJ mol^{-1} and in Å, respectively.

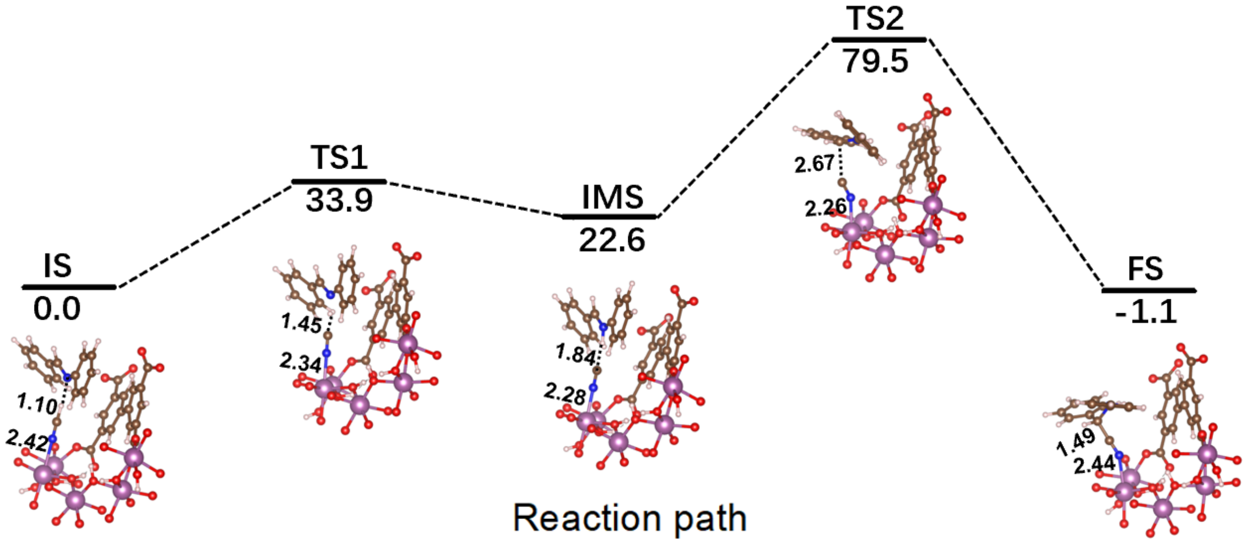

Figure S15. Potential energy profile for the reaction mechanism between N-benzylideneaniline and HCN in the MFM-300(Sc) initiated by the adsorption of HCN implying the formation of a Sc-NC complex in the first step of the reaction The initial state is taken as the references of the

potential energy. Colors code: carbon (brown), hydrogen (white), nitrogen (blue), oxygen (red) and scandium (purple). The energies and distances are reported in kJ mol-1 and in Å, respectively.

S5. References

[1] I. A. Ibarra, S. Yang, X. Lin, A. J. Blake, P. J. Rizkallah, H. Nowell, D. R. Allan, N. R. Champness, P. Hubberstey, M. Schröder, Chem. Commun. **2011**, 47, 8304.

[2] S. J. Bae, Y. M. Ha, Y. J. Park, J. Y. Park, Y. M. Song, T. K. Ha, P. Chun, H. R. Moon, H. Y. Chung, *Eur. J. Med. Chem.* **2012**, *57*, 383–390.

[3] J. P. Perdew, K. Burke and M. Ernzerhof, *Phys. Rev. Lett.*, **1996**, **77**, 3865–3868.

[4] P. E. Blöchl, *Phys. Rev. B*, 1994, **50**, 17953–17979.

[5] G. Kresse, J. Furthmüller, *Comput. Mater. Sci*. **1996**, 6, 15–50.

[6] D. Joubert, *Phys. Rev. B - Condens. Matter Mater. Phys.*, 1999, **59**, 1758–1775

[7] S. Grimme, J. Antony, S. Ehrlich, H. Krieg, *J. Chem. Phys*. **2010**, 132, 0–19.

[8] G. Henkelman, B. P. Uberuaga and H. Jónsson, *J. Chem. Phys.*, 2000, 113, 9901–9904.

[9] G. Henkelman "Vasp TST tools". http://theory.cm.utexas.edu/vtsttools/.
